# Study of Oostatic Peptide Uptake and Metabolism in Developing Ovaries of the Flesh Fly, *Neobellieria bullata*

**DOI:** 10.1673/031.010.4801

**Published:** 2010-05-17

**Authors:** Blanka Bennettová, Jiřina Slaninová, Věra Vlasáková, Jan Hlaváček, Josef Holík, Richard Tykva

**Affiliations:** ^1^ Institute of Entomology, Academy of Sciences of the Czech Republic, 370 05 České Budějovice, Czech Republic; ^2^ Institute of Organic Chemistry and Biochemistry, Academy of Sciences of the Czech Republic, 166 10 Prague 6, Czech Republic; ^3^ Institute of Experimental Botany, Academy of Sciences of the Czech Republic, 142 20 Prague 4, Czech Republic

**Keywords:** Diptera, Sarcophagidae, follicular cells, oostatic peptide, *in vivo* uptake, *in vitro* uptake, metabolites

## Abstract

The uptake and metabolism of the oostatic pentapeptide analogue of trypsin modulating oostatic factor (TMOF), H-Tyr-Asp-Pro-Ala-Pro-OH (5P), in ovaries of *Neobellieria bullata* (Parker) (Diptera: Sarcophagidae) were analyzed during their developmental stages. During selected stages of yolk deposition, the fate of [_3_HPro^3^]5P after its *in vivo* injection was compared to its uptake after *in vitro* incubation of dissected ovaries. The ovaries were analyzed from 30 s to 180 min after incubation. A detection sensitivity of 60–100 fmol of the labeled 5P was achieved using radio-high performance liquid chromatography. While the uptake of the applied radioactivity strongly depended on the stage of vitellogenesis, especially for the *in vitro* experiment, degradation of 5P was very quick and independent of whether the label was injected or incubated with the ovaries, regardless of the developmental stage of ovaries. No tracers of 5P were detected at 30 s after applying the labeled 5P in all tests.

## Introduction

Insect ovaries undergo periodic changes during the course of their development. Polytrophic ovaries of flies pass through 14 developmental stages based on the deposition of yolk in their oocytes (King 1970). From the 1^st^ to the 6^th^ stage they contain no yolk; in stages 7–10 the growing oocytes continuously fill with yolk filling up to half of the egg chamber volume. In this process, the follicular cells that form a single layer surrounding the oocyte and nutritive cells play an important role. Nutrients (proteins, peptides, lipidic substances, and carbohydrates) are synthesized and stored in the fat body from which they are transported to the growing ovaries by the hemolymph. During previtellogenesis, the tightly packed cells of the follicular epithelium shrink under hormonal influence (Sevala and Davey 1993; Davey 2000; Pszczolkowski et al. 2005) and form intercellular spaces where cytoskeletal structures and protuberancies appear (Telfer et al. 1982; Fleig 2001). This patency of follicular cells enables the transport of nutrients to the growing oocyte and takes part in the synthesis of yolk components in the oocyte cytoplasm (Huebner et al. 1975; Kelly and Telfer 1979; Brennan et al. 1982; Fausto et al. 2005). The activity of follicular cells may vary with the demand of particular nutrients by the oocyte during a reproductive cycle (Davey 1996).

**Figure 1.**
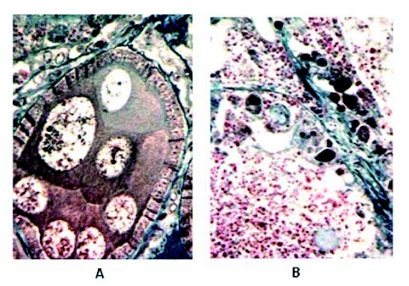
Histology of degenerative changes in the flesh fly *Neobellieria bullata* ovaries after application of oostatic pentapeptide (5P). A: Normal ovary development. B: The inner space of egg chamber filled up with nuclei and cells that originated from follicular epithelium. High quality figures are available online.

A study using the trypsin modulating oostatic factor (TMOF) of the mosquito, *Aedes aegypti* L. (Diptera: Culicidae), and the flesh fly, *Neobellieria bullata* (Parker) (Diptera: Sarcophagidae), for the investigation of their sterilizing effect on a partly autogenous strain of *N. bullata* had negative results, and, consequently, a new study was done of peptides with the C-terminal shortened sequence of Aed-TMOF (H-Tyr-Asp-Pro-Ala-PrO6-OH; Borovsky et al. 1990, 1994). The evaluation of morphological changes was done on the structures of the first and second egg chambers of *N. bullata* during the reproductive cycle. The greatest effects on developing ovaries were found after injection of the respective pentapeptide 5P (H-Tyr-Asp-Pro-Ala-Pro-OH) or tetrapeptide 4P (H-Tyr-Asp-Pro-Ala-OH) (Slaninová et al. 2004). In studies of the effects of 5P on vitellogenic stages (Hlaváček et al. 1997, 1998; Bennettová et al. 2002; Slaninová et al. 2004) differences were found between morphological changes of both egg chambers: in the first one there were no visible effects, while in the second, proliferation of the follicular epithelium into the inner space of egg chamber, followed by resorption was observed (Figure 1). Therefore, its ^3^H-labeled forms were used for further studies on radioactivity accumulation and degradation of the oostatic peptides in the flesh fly *N. bullata* and other insects (Tykva et al. 1999, 2007; Slaninová et al. 2004; Hlaváček et al. 2007). On the other hand, the native TMOF of *N. bullata*, hexapeptide H-Asn-Pro-Thr-Asn-Leu-His-OH (Bylemans et al. 1994; de Loof et al. 1995), has no structural similarity to the above oostatic peptides and lacks any oostatic effects, nor does its isosteric analogue H-Asn-Proψ[CH_2_O]-D-Thr-Asn-Leu-His-OH, (Bennettová, unpublished results; Hlaváček et al. 2004). Oostatic peptides represent an effective tool for insect control by inhibiting egg development (Slaninová et al. 2004; Tykva et al. 2007).

Relative to other biologically active substances (e.g. pesticides, fungicides or juvenogens), oostatic peptides are relatively simple to synthesize and have no negative environmental impact (Tykva et al. 2004). They are also soluble in water. However, their mode of action is not understood.

In this study, the uptake of an *in vivo* injected oostatic pentapeptide (5P) into ovaries of *N. bullata* was estimated at different stages of vitellogenesis (7–10) using radiolabeled peptide. Isolated ovaries were also incubated with radiolabeled peptide *in vitro.* The radioactive metabolites in the ovaries after *in vivo* and *in vitro* uptake of radiolabeled peptide were compared.

## Materials and Methods

### Radiolabeled peptide and developmental stages

Tritiated oostatic pentapeptide (5P) H-Tyr-Asp-[^3^H]Pro-Ala-Pro-OH, [^3^HPrO^3^] 5P, 1.44TBq/mmol, with radiochemical purity > 98%, as described earlier Hlaváček et al. (2007) was used in the study. Different stages of yolk deposition were classified according to the scale developed by King (1970). For *in vivo* applications the ovaries were divided into 2 groups (one with eggs in the 7^th^ and 8^th^ stages of development and the second with eggs in the 9^th^ and 10^th^ stages of development). For the experiments *in vitro*, each of these four tested stages of egg development were evaluated individually.

### *In vivo* experiments

[^3^HPro^3^]5P was injected in 5 µl (37 kBq) of physiological solution into left upper part of the thoraces of ether-anesthetized female *N. bullata*. Flies were then dissected at given time intervals, and 12 pairs of ovaries of the same developmental stage (7, 8, 9 and 10) were selected for each interval. Each pair of ovaries was placed into a separate scintillation vial and covered with 0.5 ml of tissue solubilizer (NCS II, Amersham International, www.gelifesciences.com). After six days, 10 ml of liquid scintillator EcoLite (ICN Biochemicals Inc.) were added and the radioactivity determined in the spectrometer Beckman 6500. The highest and lowest values of the sets were eliminated and the 10 remaining samples were used for evaluation of the mean values and their standard deviations. Three such experiments were carried out independently and from all of them the total mean values with their standard deviations (from ± 16% to ± 25%) were calculated. For metabolite determination by radio-HPLC, in each time interval 10–15 pairs of ovaries were dissected from the injected *N. Bullata*, pooled into groups according to developmental stage and frozen at -70° C until extraction.

### *In vitro* experiments

Twelve dissected pairs of ovaries of identical stage and appearance were placed into a solution of [^3^HPro^3^]5P (555 kBq in 450 µl of physiological solution) in small embryo dishes at room temperature. At time periods equal to those for the *in vivo* experiments, the ovaries were removed, washed twice in physiological solution, and each pair was placed into individual scintillation vials for determination of total radioactivity and treated as described for the *in vivo* experiment. From three independent experiments, the total S.D. was calculated from ± 13% to ± 24%. Simultaneously, incubation for the selected time period was done for metabolite determination, and the sample was frozen as for the *in vivo* experiments.

### Extraction for metabolite determination

An ice-cold solution (0.4 ml) of protease inhibitor cocktail Complete Mini (Roche Applied Sciences, www.roche-applied-science.com) (1 tablet dissolved in 3.5 ml of 50 mM HEPES buffer pH 7.6) was added to the frozen pooled ovaries in an Eppendorf tube, and the contents were homogenized for 1 min using a Teflon pestel. After centrifugation, the supernatant was removed and either immediately analyzed by radio-HPLC or frozen and analyzed later.

### Analysis of 5P metabolites

All radio-HPLC analyses were performed using a Waters liquid Chromatograph (Waters, www.waters.com). A programmable UV detector was connected on-line to a radiometric flow-through detection system (Beckman 171, www.beckman.com). The stainless steel analytical column (250 × 4 mm) LiChroCART (Merck, www.merck.com), packed with LiChrosphere WP-300, with a particle size of 5 µm was used. The column was protected with a (4 mm × 4 mm) guard column packed with LiChrosphere 100 RP-18, particle size 5 µm (Merck). The mobile phase was composed of the aqueous phase (0.035% TFA in redistilled water) and the organic phase (0.05% in acetonitrile). After passing through the UV detector, the eluent was continuously mixed with the liquid scintillator Ready Safe (Beckman Coulter) with a ratio of 1:2.5 (v/v) in an on-line mixer. The mixture was run through a 500 µl detection cell. The radiometric detector threshold was set at 0.02%. The UV detector was set at 230 nm, 0.05 AUFS. Separation was performed at ambient temperature using a 30 min linear gradient from 0% to 30% organic phase using a flow rate of 0.8 m1/min and continuous degassing with helium. A sample volume of 20 to 80 µl was used. The area of each peak was evaluated as the ratio of its counting rate to the totally measured counting rate in all peaks of the appropriate radiochromatogram (relative concentration crel in percentage). The stability of the [^3^HPro^3^]5P was checked before each experiment.

Samples were centrifuged for 5 min, and an aliquot of the supernatant was analyzed. Standard unlabeled peptides (Hlaváček et al. 2007) as well as non-active proline were detected by UV, and their retention times were compared to that of the peaks in the radiochromatogram. The retention times of radioactive fractions were corrected to the time delay between the UV and the radiometric detector (0.55 min). The precision of the method was expressed as the coefficient of variation in percentage that varied from 1.3 to 7.7%). It was determined by analyzing five replicates of the same biological sample within one day.

The extraction procedure recovery was evaluated using [^3^HPro^3^]5P calibration solutions of three different concentrations (42, 150 and 370 kBq/ml) by comparison of the extracted and the applied radioactivity. The average recovery was between 86.6% and 97.2% (*n* = 4) with precision range from 3.3 to 5.2 (% coefficient of variation). The linearity of the radiometric detector response was verified using [^3^HPro^3^]5P calibration solutions in the range of 0.2–10 kBq with an average correlation coefficient of 0.997 (*n* = 4). The absolute detection limit in the system, defined by a signal-to-noise ratio of 3, was assigned for the 5P in the range of 85–150 Bq that corresponds to 59–104 (rounded 60–100) fmol.

**Figure 2.**
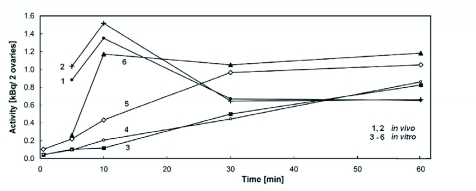
Radioactivity of ovaries in different vitellogenic stages of development in dependence on time after application *in vivo* (1, 2) or *in vitro* (3–6). Stage of development according to King (1970): 1: 7th and 8th; 2: 9th and 10th; 3; 7th; 4: 8th; 5: 9th; 6: 10th. Sets of 10 samples in 3 independent experiments were evaluated for each point, S.D. of the presented means from ± 16% to ± 25%. High quality figures are available online.

## Results and Discussion

**Figure 3.**
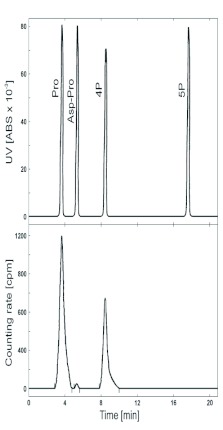
Lower; radiochromatogram of an extract of ovaries of the 10th stage 5 min after [^3^HPro^3^]5P *in vitro* application; Upper; chromatogram of non-active standards. High quality figures are available online.

In this study, attention was focused on the uptake of the oostatic pentapeptide 5P (H-Tyr-Asp-Pro-Ala-Pro-OH) by ovaries of *N. bullata*. As a continuation of previous studies (Tykva et al. 2007), the uptake was monitored in relation to the stage of egg development (vitellogenesis or yolk deposition) and tested *in vitro*. The total radioactivity in the ovaries (Figure 2) was determined, and the radioactive components in extracts of ovaries were analyzed. Radiolabeled metabolites of [^3^H-Pro^3^]5P found in ovaries were identified using synthetic standards of non-labeled sequences (Hlaváček et al. 2007) as illustrated in Figure 3.

As can be seen in Figure 2, the time course of radioactivity uptake was different for *in vitro* and *in vivo* experiments. In the latter case, the radioactivity increased until 10 min after application, then decreased, and after 30 min it was almost constant. No statistically significant differences were found between the two experimental groups (curves 1 and 2 in Figure 2). Such a time coarse could be explained by a single injection of [^3^HPro^3^]5P. On the contrary, for the *in vitro* experiments, radioactivity continuously increased because there was permanent contact of 5P with the ovaries. Nevertheless, there were statistically significant differences between the two following groups. With small yolk deposition (Figure 2, curves 3 and 4), the radioactivity increased slowly and continuously during the measured time interval. In the later developmental stages (Figure 2, curves 5 and 6), a rapid increase was found until 30 min, at which time the radioactivity was practically continuous, which was similar to the results of the *in vivo* experiment. In this time interval of practically stable radioactivity, the concentration of the 5P metabolites seemed to reach its maximum.

Independently of application method, the metabolites were qualitatively identical, and no 5P was found by 30 s after application (Table 1). This finding suggests an effective enzymatic system for peptide degradation. Such system may be located in the interfollicular spaces once their patency was evoked by a juvenile hormone or some other hormonal action (Davey 2000), on the oocyte membrane adjacent to the apical part of the follicular cells (Telfer et al. 1982), or possibly in the oocyte cytoplasm. The process of follicular cell patency is finished prior to the onset of vitellogenesis (Telfer at al. 1982). The active peptide intake increases with continuous yolk deposition even though a receptor responsible for oostatic peptide transport was not previously found (Slaninová et al. 2004).

Regarding the metabolites, the composition of radioactive substances was qualitatively almost identical during the entire period followed (30 s-180 min), both after incubation with the peptide *in vitro* and also after administration of the peptide *in vivo*. As can be seen in Table 1, there was a very rapid degradation of the 5P, which was not detectable in either case after 30 s. The same results were found for all tested stages during egg development. These results suggest that there is an extremely rapid metabolic cleavage of the N-terminal Tyr^1^, and the remaining 4P (H-Asp-[^3^H]Pro-Ala-Pro-OH) probably is then degraded into two dipeptides from which H-Asp-[^3^HPro]-OH then gives rise very quickly to [^3^H]Pro.

**Table 1.**
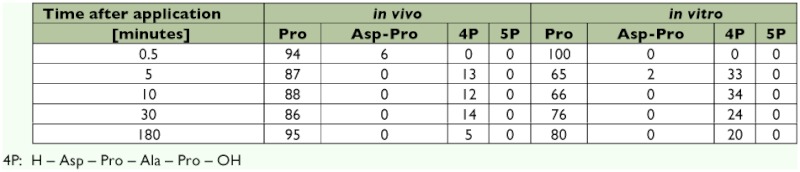
Metabolites of [^3^H- Pro^3^]SP in ovaries of stage 10 in % of total radioactivity

The differences between the *in vivo* and *in vitro* experiments may be explained by the partly metabolized 5P (*in vivo*), which is incorporated into the fat body. Then, as with other nutrients, it is delivered by hemolymph to the ovaries (Tykva et al. 2004). On the contrary, the *in vitro* intake of non-metabolized 5P begins in the incubated ovaries, where only the 5P alone is available and the metabolism of ovaries takes place without any nutrient.

Very interesting is the fact that isolated ovaries with no connection to the tracheal system, the nervous system and/or the hemolymph were still able to use the 5P by a way other than simple diffusion and and to metabolize it to qualitatively identical metabolites as occured in intact ovaries. Previous studies revealed rich innervation of ovaries by branches of the median nerve that originate in thoracic ganglium, and, via the lateral oviduct, innervate each ovariole along the ovarian sheath (Bennettová-Řežábová 1971). Neurosecretory cells were located in close connection with follicular cells and granules of neurosecretory material were present (Bennettová and Mazzini 1989). Such findings might be responsible for the independent functioning of ovaries *in vitro*, at least for a limited amount of time.

### Conclusions

These results showed that the uptake of the oostatic 5P metabolites into the ovaries of *N. bullata* depended on the stage of the vitellogenic ovaries. Unlike the case after *in vivo* injection, the *in vitro* experiments show that ovaries that have low amounts of yolk also accumulate the metabolites at a slower rate. On the other hand, in later vitellogenic stages radioactivity quickly reached a maximum and then stayed almost constant, which was similar to the *in vivo* assay. In all tested ovaries, no 5P was found by 30 s after application, and the same 5P radiometabolites were detected. The results of these analyses of [^3^HPro]5P and its metabolites seem to point towards the existence of an enzymatic system that very effectively degrades the 5P TMOF analogue during the transport into the egg chamber. An active intake of the analyzed sequences into the intercellular spaces between follicular cells may be assumed.

## Abbreviations

HPLC: high performance liquid chromatography;
TMOF: trypsin modulating oostatic factor

## References

[bibr01] Bennettová-Řežábová B (1971). The regulation of vitellogenesis by the central nervous system in the blow-fly *Phormia regina* (Meigen).. *Entomologica Bohemoslovaca*.

[bibr02] Bennettová B, Mazini M, Bennettová B, Soldán T, Gelbič I (1989). The inervation of female reproduction tract of *Phormia regina*.. *Proceedings of International Conference on Regulation of Insect Reproduction IV, 1987.*.

[bibr03] Bennettová B, Hlaváček J, Tykva R (2002). Follicular epithelium changes after application of insect oostatic hormone analogues in *Neobellieria bullata* (Diptera).. *Abstracts of VIIth European Congress of Entomology* 2002. Abstract No. 10.

[bibr04] Borovsky D, Carlson DA, Griffin PR, Shabanovicz J, Hunt DF (1990). Mosquito oostatic factor: A novel decapeptide modulating trypsin-like enzyme biosynthesis in the midgut.. *Federation of American Societies for Experimental Biology Journal*.

[bibr05] Borovsky D, Song Q, Ma M, Carlson DA (1994). Biosynthesis, secretion and cytoimmunocytochemistry of trypsin modulating oostatic factor of *Aedes aegypti*.. *Archives of Insect Biochemical Physiology*.

[bibr06] Brennan MD, Weiner AJ, Goralski TJ, Mahowald AP (1982). The follicle cells are a major site of vitellogenin synthesis in *Drosophila melanogaster*.. *Developmental Biology*.

[bibr07] Bylemans D, Borovsky D, Hunt DF, Shabanowitz J, Granwels L, DeLoof A (1994). Sequencing and characterization of trypsin modulating oostatic factor (TMOF) from the ovaries of the gray flesh fly *Neobellieria (Sarcophaga) bullata*.. *Regulatory Peptides*.

[bibr08] Davey KG (2000). The modes of action of juvenile hormones: Some questions we ought to ask.. *Insect Biochemistry and Molecular Biology*.

[bibr09] Davey KG (1996). Hormonal control of the follicular epithelium during vitellogenium uptake.. *Vertebrate Reproduction and Development*.

[bibr10] Davey KG, Locke M, Smith DS (1980). Hormonal-regulation of vitellogenesis in *Rhodnius prolixus*.. *Insect Biology in the Future “WBW 80”.*.

[bibr11] De Loof DA, Bylemans D, Schoofs L, Janssen I, Huybrechts R (1995). The folliculostatins of two dipteran insect species, their relation to matrix proteins and prospects for practical applications.. *Entomologia Experimentalis et Applicata*.

[bibr12] DeLoof DA, Bylemans D, Schoofs L, Janssen I, Spittaels K, Van den Broeck J, Huybrechts R, Borovsky D, Hue Y, Koolman J, Sower S (1995). Folliculostatins, gonadotropins and a model for control of growth in the gray flesh fly *Neobellieria (Sarcophaga) bullata*.. *Insect Biochemical Molecular Biology*.

[bibr13] Fausto AM, Gambellini G, Marrini M, Cecchettini A, Giorgi F (2005). Yolk uptake through the follicle epithelium in the ovary of the stick insect *Carausius morosus*.. *Arthropod Structure and Development*.

[bibr14] Fleig R (2001). Ultrastructure of follicle cells and yolk uptake of oocytes in vitellogenic follicles of cotton bug *Dysdercus intermedius* (Heteroptera: Pyrrhocoridae) and the honeybee *Apis mellifera* (Hymenoptera: Apidae).. *Entomologia generalis*.

[bibr15] Hlaváček J, Bennettová B, Barth T, Tykva R (1997). Synthesis, radiolabeling and biological activity of peptide oostatic hormone and its analogues.. *Journal of Peptide Research*.

[bibr16] Hlaváček J, Tykva R, Bennettová B, Barth T (1998). The C-terminus shortened analogs of the insect peptide oostatic hormone with accelerated activity.. *Bioorganic Chemistry*.

[bibr17] Hlaváček J, Černý B, Bennettová B, Holík J, Tykva R (2007). Preparation of tritiated oostatic peptides for study of radioactivity incorporation in flesh fly *Neobellieria bullata*.. *Amino Acids*.

[bibr18] Huebner E, Tobe SS, Davey KG (1975). Structural and functional dynamics of oogenesis in *Glossina austeni-*gentral features, previtellogenesis and nurse cells.. *Tissue Cell*.

[bibr19] Kelly TJ, Telfer WH (1979). Function of the follicular epithelium in vitellogenic oncopeltus follicles.. *Tissue Cell*.

[bibr20] King RC (1970). Ovarian Development in *Drosophila melanogaster*..

[bibr21] Pszczolkowski MA, Peterson A, Srinivasan A, Ramaswamy SB (2005). Pharmacological analysis of ovarial patency in *Heliothis virescens*.. *Journal of Insect Physiology*.

[bibr22] Němec V, Tykva R, Hlaváček J, Holík J (2007). Sterilizing effect of an oostatic pentapeptide on reproduction of *Pyrrhocoris apterus*.. *Collection Symposium Series*.

[bibr23] Sevala VL, Davey KG (1993). Juvenilehormone dependent phosphorylation of a 100 KDA polypeptide is mediated by protein-kinase C in the follicle cells of *Rhodnius prolixus*.. *Invertebrate Reproduction and Development*.

[bibr24] Slaninová J, Bennettová B, Nazarov E, Šimek P, Holík J, Vlasáková V, Hlaváček J, Černý B, Tykva R (2004). Activity and mechanism of action of insect oostatic peptides in flesh fly.. *Bioorganic Chemistry*.

[bibr25] Telfer W, Huebner E, Spencer DS, King RC, Akai H (1982). The cell biology of vitellogenic follicles in *Hyalophora* and *Rhodnius*.. *Insect Ultrastructure*.

[bibr26] Tykva R, Hlaváček J, Němec V, Bennettová B, Del Re AAM, Brown C, Capri E, Errera G, Evans SP, Trevisan M (1999). Effect of a peptide and some of its analogs on the reproduction of *Sarcophaga bullata* and *Locusta moratoria*.. *Proceedings of 11^th^ Symposium on Pesticide Chemistry, 1999.*.

[bibr27] Tykva R, Vlasáková V, Novák J, Havlíček L (2004). Radio high-performance liquid chromatography for ecotoxicity assessment of insect growth regulators.. *Journal of Chromatography A*.

[bibr28] Tykva R, Bennettová B, Vlasáková V, Holík J, Šimek P, Černý B, Hlaváček J, Slaninová J, Flegel M, Fridkin M, Gilon Ch, Slaninová J (2004). Degradation of active analogues of insect oostatic decapeptide.. *Peptides 2004. Proceedings of the 3^rd^ International and 28th European Peptide Symposium*.

[bibr29] Tykva R, Hlaváček J, Vlasáková V, Černý B, Borovičková L, Bennettová B, Holík J, Slaninová J (2007). Radiochromatographic assay of metabolites of the oostatic peptide labeled in different positions of the peptide chain.. *Journal of Chromatography B*.

